# Ex Vivo Pharmacokinetic/Pharmacodynamic Integration Model of Cefquinome Against *Escherichia coli* in Foals

**DOI:** 10.3390/vetsci12040294

**Published:** 2025-03-22

**Authors:** Tiantian Gao, Xuesong Liu, Di Qiu, Yanan Li, Zongsheng Qiu, Jingjing Qi, Shuxin Li, Xiaoyan Guo, Yan Zhang, Ziqi Wang, Xiang Gao, Yuhui Ma, Tianwen Ma

**Affiliations:** 1 Heilongjiang Key Laboratory for Laboratory Animals and Comparative Medicine, College of Veterinary Medicine, Northeast Agricultural University, Harbin 150030, China; 13009860872@163.com (T.G.); qd13836005047@163.com (D.Q.); 13796685756@163.com (Y.L.); 2108731648@163.com (Z.Q.); 18020189739@163.com (J.Q.); lishuxin2000@yeah.net (S.L.); 13673051709@163.com (X.G.); gam2006gx@neau.edu.cn (X.G.); 2 Heilongjiang Province Key Laboratory of Veterinary Drugs, Branch of Animal Husbandry and Veterinary of Heilongjiang Academy of Agricultural Sciences, Qiqihar 161005, China; abliuxuesong@yeah.net (X.L.); zhangyanyan008@163.com (Y.Z.); 3 Feihe (Qiqihar) Dairy Co., Ltd., Qiqihar 161000, China; ziqi_wang@foxmail.com; 4 Zhaosu County Xiyu Horse Industry Co., Ltd., Zhaosu County, Yili 835699, China

**Keywords:** cefquinome, *Escherichia coli*, PK/PD model, dose regimen

## Abstract

Cefquinome is used to treat septicemia caused by *Escherichia coli* (*E. coli*) and respiratory infections caused by *Streptococcus equi* subsp. *zooepidemicus* in foals. However, studies reporting the use of cefquinome to target *E. coli* as pathogens of sepsis are lacking. Therefore, this study aimed to determine the optimal dosage regimen for cefquinome against *E. coli* using a PK/PD model. After the administration of 1 mg/kg cefquinome (intramuscularly or intravenously), blood samples were collected at different time points to determine the serum concentration of cefquinome via HPLC. The pharmacokinetic parameters were evaluated via NCA (WinNonlin 5.2.1 software). The main pharmacokinetic parameters of cefquinome in foals were as follows: after intravenous administration, the elimination half-life (T_1/2β_) was 2.35 h, the area under the curve (AUC_0–last_) was 12.33 μg·h/mL, the mean residence time (MRT_0–last_) was 2.67 h, and the clearance rate (CL) was 0.09 L/h/kg. After intramuscular administration, the peak concentration (C_max_) was 0.89 μg/mL, the time to reach the maximum serum concentration (T_max_) was 2.16 h, T_1/2β_ was 4.16 h, AUC_0–last_ was 5.41 μg·h/mL, MRT_0–last_ was 4.92 h, CL was 0.15 L/h/kg, and the absolute bioavailability (F) was 43.86%. An inhibitory sigmoid Emax model was used to integrate the PK/PD indices with ex vivo antimicrobial effects to identify pharmacodynamic targets (PDTs). According to the dose calculation formula, the doses of intramuscularly administered cefquinome required to achieve bacteriostatic effects, bactericidal effects, and bactericidal elimination were 1.10, 1.66, and 2.28 mg/kg, respectively. However, further studies are warranted to verify the therapeutic efficacy of cefquinome in clinical settings.

## Simple Summary

Bacterial infection is a major cause of death in foals. *Escherichia coli* (*E. coli*) is a Gram-negative bacterium that causes sepsis, which can be life-threatening in horses. Cefquinome has broad-spectrum antimicrobial activity and is widely used in the treatment of animal diseases. At the same time, it is the fourth generation of cephalosporin antibiotics for veterinary use, and it has good antibacterial activity against both Gram-positive and Gram-negative bacteria. Cefquinome is utilized for the treatment of septicemia induced by *E. coli*, as well as respiratory infections caused by *Streptococcus equi* subsp. *zooepidemicus* in foals. However, there is a lack of studies using cefquinome to target *E. coli* as a pathogen of sepsis. Pharmacokinetic/pharmacodynamic (PK/PD) modeling is a fundamental step in semi-mechanical methodology aimed at improving dose planning of systemically active antimicrobial agents. PK/PD models can determine the relationship between drug concentration, antibacterial action, and time, deriving the most effective dosing regimen. This study was based on this model to verify the optimal dosing regimen for *E. coli* infection in foals and to provide a theoretical basis for the precision treatment of foals.

## 1. Introduction

During the initial weeks of life, bacterial infection is a leading cause of death in foals [1]. Bacterial infection can progress to life-threatening septicemia, with *Escherichia coli* (*E. coli*), a Gram-negative bacterium, being the commonly identified organism [2,3]. As a fourth-generation cephalosporin antibiotic used only in veterinary medicine, cefquinome exerts good antibacterial effects against various bacteria, including Gram-positive and Gram-negative bacteria [4,5]. Cefquinome exhibits broad-spectrum antibacterial activity and is widely used in the treatment of animal diseases. Mechanistically, it inhibits the synthesis of bacterial cell wall proteins to cause bacterial death [6,7]. Cefquinome is used to treat respiratory diseases caused by *Streptococcus equi* subsp. *zooepidemicus* in horses and septicemia caused by *E. coli* in foals. The recommended dosage regimen is 1 mg/kg [8]. A benefit of using cefquinome to treat septicemia is its lower risk of causing nephrotoxicity in foals whose renal function is impaired or unknown [9].

Pharmacokinetic/pharmacodynamic (PK/PD) modeling serves as a foundational step in semi-mechanistic methodology aimed at improving dosage plans for systemically active antimicrobial medication [10]. The PK/PD model can determine the relationship among drug concentrations, antimicrobial action, and time. Through detailed calculations, the model can suggest the most effective dosage regimen [11]. PK/PD models are constructed by integrating specific pharmacokinetic (PK) parameters, such as the area under the curve (AUC) and peak concentration (C_max_), as well as pharmacodynamic (PD) parameters, such as time–kill curves. The main indices of PK/PD models include the area under the curve-to-the MIC ratio (AUC/MIC), the percentage of time during the dosing interval in which the drug concentration exceeded the MIC (%T > MIC), and the peak drug concentration-to-the MIC ratio (C_max_/MIC). In particular, the AUC/MIC ratio and %T > MIC are two indices frequently used to evaluate the efficacy of veterinary medications. Due to the fact that the AUC/MIC index has a time dimension, dividing AUC_0–24h_/MIC by 24 h, which results in an index without a time dimension, is therefore recommended for use [10]. PK/PD modeling offers numerous benefits compared to dose titration studies. Unlike dose titration studies that necessitate the examination of various doses, PK/PD modeling enables the establishment of a projected dosage regimen through the analysis of only one dose [12].

In past research on the health and disease treatment of foals, numerous scholars have carried out in-depth explorations on various diseases, enriching our understanding of the physiological functions and disease mechanisms of foals to a certain extent. However, there are still many gaps in the current research regarding the diseases caused by *E. coli* infection in foals, especially on how to develop targeted dosing regimens through precise PK/PD models. Although the current research results involve the treatment of *E. coli* infection in foals, there is still a lack of systematic and in-depth studies on the precise control of dosage and the impact of individual differences on drug metabolism and efficacy. Most of the studies remained only at the recommendation level of empirical medication, failing to fully combine the principles of PK and PD and develop a scientific and precise dosing regimen according to the unique physiological characteristics of foals. This lack of research makes it difficult for equine veterinarians to determine the optimal dose for the actual treatment of *E. coli* infection in foals, which may affect the treatment effect and even lead to bacterial resistance problems.

Although previous studies have reported the use of PK/PD models to evaluate the efficacy of cefquinome in certain animals, these models have not been used to design optimized dosage regimens for targeting *E. coli* in foals. This report presents a PK/PD model developed based on the pharmacokinetics of cefquinome and its minimum inhibitory concentration (MIC) against *E. coli* isolated from foals, with the aim of optimizing the dosage regimen.

## 2. Materials and Methods

### 2.1. Drug Reagents and Organisms

Cefquinome sulphate in a sterile powder form was obtained from Dr. Ehrenstorfer (Augsburg, Germany). Acetonitrile and methanol were obtained from Sigma (St. Louis, MO, USA). Trifluoroacetic acid (TFA) and other HPLC-grade chemicals were obtained from Aladdin (Shanghai, China). Cefquinome sulphate injection was obtained from Qilu Animal Health Products Co., Ltd. (Jinan, China). All reagents used in this study were of HPLC grade.

The pathogenic *E. coli* strain used in this study was previously isolated from a foal with septicemia in Heilongjiang Province, China. This strain was used for PD assessment. Additionally, a fully characterized *E. coli* strain, ATCC 25922, was obtained from the China Institute of Veterinary Drug Control (Beijing, China). All strains were stored in a freezer (−80 °C) until required.

### 2.2. Animals and Experimental Design

Animal experiments were performed on 10 Ili foals aged between 7 months and 1 year, with an average weight of 191 ± 21.7 kg. These foals were provided by Zhaosu County Xiyu Horse Industry Co., Ltd. (Yili, China). All foals were acclimatized to the new environment for 2 weeks before the experiments. Additionally, they were assessed for fitness (including physical examination and evaluation of heart rate and rectal temperature) before testing. None of the foals had received any medical treatment for at least 2 weeks before enrollment. All foals were kept in separate, shaded, and ventilated sheds with ad libitum access to hay and water. A total of 10 Ili foals were randomly divided into 2 groups. A two-phase crossover study was conducted, involving 10 foals. In the first phase, 5 foals in one group received 1 mg/kg of cefquinome intramuscularly in the cervical region (i.e., between the scapula, cervical spine, and posterior cervical ligament), whereas 5 foals in the other group received 1 mg/kg of cefquinome intravenously (IV) via the jugular vein. After a 2-week interval to ensure complete drug excretion, the second phase commenced, and the treatments of the two groups were reversed. After cefquinome was administered, blood samples were obtained from the left jugular vein at the following time points: 0.083, 0.167, 0.25, 0.5, 0.75, 1, 2, 3, 6, 9, 12, and 24 h. The samples were collected in tubes without anticoagulants. To extract serum, the samples were centrifuged at 3500× *g* for 10 min at 4 °C. The serum samples were stored at −80 °C until further use.

### 2.3. HPLC Analysis for Cefquinome Detection

The serum concentration of cefquinome was determined via HPLC, as described by Lee et al., with slight modifications [8]. For the gradient elution, 0.1% TFA aqueous solution was used as mobile phase A, and acetonitrile was used as mobile phase B. The mobile phase conditions for HPLC analysis of cefquinome were as follows: the flow rate was maintained at 1.0 mL/min throughout the 15 min run. The gradient program started with 90% mobile phase A and 10% mobile phase B at 0 min. At 7 min, the composition changed to 50% mobile phase A and 50% mobile phase B, which was held at this ratio until 10 min. The gradient returned to the initial conditions at 11 min and was maintained until the end of the run at 15 min. The ShimNex CS C18 column (id, 250 mm × 4.6 mm; particle size, 5 μm; Shimadzu Co., Kyoto, Japan) was used for separation. The HPLC system consisted of two LC-20AD pumps controlled by a CBM-20A system controller, a degasser (DGU-20A3R) for mobile phase degassing, an autosampler (SIL-20A) for sample introduction and a SPD-M20A diode array detector to monitor absorbance at 268 nm. This system allowed for the precise quantification and analysis of cefquinome levels in foal serum samples.

To prepare samples for HPLC, 300 µL of methanol was added to 300 µL of serum, and the mixture was vortexed for 2 min. Subsequently, the mixture was centrifuged at 8500× *g* for 15 min, and the supernatant was collected. A total of 20 μL of each supernatant sample was injected into the HPLC system for analysis.

### 2.4. Pharmacokinetic Analysis of Cefquinome

The primary PK parameters were calculated using a non-compartmental model in WinNonlin (version 5.2.1) software (Pharsight, Mountain View, CA, USA). These parameters included the elimination half-life (T_1/2β_), peak serum concentration (C_max_), time to reach the maximum serum concentration (T_max_), area under the curve (AUC_0–last_), and mean retention time (MRT_0–last_).

### 2.5. Determination of MIC

The *E. coli* strain ATCC25922 was used for quality control. The MIC was determined following the guidelines established by the Clinical Laboratory Standards Institute. Briefly, at least 10 freshly cultured colonies were transferred into Mueller Hinton Broth (MHB) and incubated at 37 °C. The suspension was adjusted with sterile saline until its turbidity matched that of the 0.5 McFarland standard. The colonies were counted to confirm the inoculum size. The bacterial cultures were diluted to approximately 2 × 10^6^ CFU/mL, and 100-µL aliquots of the cultures were added to a 96-well plate to prepare a series of 2-fold dilutions of the drug concentrations. To enhance the accuracy of the assessment, three sets of overlapping 2-fold dilutions were used (0.013–12.8, 0.014–13.83 and 0.016–16 μg/mL). The MIC was defined as the lowest concentration of cefquinome that inhibited the visible growth of bacteria after incubation for 24 h at 37 °C.

### 2.6. Measurement of Post-Antibiotic Effects 

The post-antibiotic effects (PAEs) of cefquinome on *E. coli* were analyzed using the drug removal method. The *E. coli* strain was incubated with cefquinome at concentrations of 1×, 2×, and 4× MIC. After 1 and 2 h of incubation, the drug was removed through repeated centrifugation in a fresh medium. Colony counts were assessed at different time points, and growth kinetic curves for bacteria were plotted to evaluate PAEs.

### 2.7. In Vitro Time–Kill Curves

Bacterial cultures in the stationary phase were mixed with 10 mL of MHB and foal serum, and the mixture was diluted to concentrations of 10^6^ CFU/mL and 10^7^ CFU/mL. Subsequently, cefquinome at different concentrations (0×, 0.5×, 1×, 2×, 4×, 8×, and 16× MIC) was introduced into the bacterial suspensions. Bacterial populations were evaluated after 1, 3, 6, 9, 12, and 24 h. Following a series of 10-fold dilutions, the samples were added onto trypticase soy agar (TSA) plates and incubated at 37 °C for 18–20 h. The minimum detection limit was determined to be 200 CFU/mL.

### 2.8. Ex Vivo Time–Kill Curves

The ex vivo time–kill curves of cefquinome against *E. coli* were generated using serum samples. Serum was collected from foals at 0.083, 0.167, 0.25, 0.5, 0.75, 1, 2, 3, 6, 9, 12, and 24 h after IM administration of cefquinome. A total of 0.5 mL of each serum sample was mixed with 50 μL of bacterial cultures in the stationary phase, resulting in the final concentration of approximately 1 × 10^6^ CFU/mL. After 1, 3, 6, 9, 12, and 24 h of incubation, 20-μL aliquots were taken from each culture tube and subjected to 10-fold serial dilution. Subsequently, 20 μL of each diluted suspension was added to the quadrants of TSA plates. The detection limit was determined to be 200 CFU/mL.

### 2.9. PK/PD Integration and Modeling

For cefquinome, the surrogate indicator of antimicrobial activity, the ratio of AUC_0–24h_/MIC divided by 24 h, was evaluated by correlating in vitro PK parameters, MIC values and ex vivo PD parameters in serum. An inhibitory sigmoid E_max_ model was used to examine the correlation between the ex vivo ratio of dividing AUC_0–24h_/MIC by 24 h and the change in bacterial counts from the baseline to the measured count after 24 h of incubation in serum. This relationship was defined by the equation presented below.

$$E=E_{max}-\frac{\left(E_{max}-E_{0}\right)\times C_{e}^{N}}{C_{e}^{N}+{EC}_{50}^{N}}$$

The effect of the antibacterial agent, denoted as E, was quantified by the change in the log_10_ difference in bacterial counts after 24 h of incubation relative to the initial log_10_ CFU/mL present in the serum sample. In the abovementioned equation, E_max_ represents the variation in the log_10_ difference in bacterial counts observed between the 0 and 24 h intervals in samples that did not receive the drug. E_0_ indicates the change in the log_10_ difference in bacterial counts for the samples treated with cefquinome. EC_50_ refers to the pharmacokinetic/pharmacodynamic (PK/PD) index of the drug that achieves 50% of its peak antibacterial effect. C_e_ represents the PK/PD index, and N represents the Hill coefficient, which indicates the steepness of the curve. The PD parameters were evaluated using GraphPad Prism software (version 8.02, San Diego, CA, USA).

### 2.10. Monte Carlo Simulation

Monte Carlo simulation involving 10,000 subjects was performed using Crystal Ball Professional V11.1.3 (Oracle, Redwood, CA, USA) in accordance with a method described by Xiao et al. [13]. This simulation was based on the PK parameters of cefquinome observed in serum following IM administration, including the PK/PD index targets obtained from ex vivo PK/PD modeling and the MIC distribution of cefquinome against 53 *E. coli* strains collected during this study. The PK parameter AUC_0–24h_ was assumed to follow a log-normal distribution, represented by mean values and confidence intervals. Considering the distribution of MIC values, the ratio of AUC_0–24h_/MIC by 24 h was computed using the Monte Carlo simulation. Additionally, the target attainment rate (TAR) for the determined dose was assessed based on the distribution of MIC.

### 2.11. Estimation of Dosage Regimen

The dose required to achieve different antibacterial effects was determined based on an equation reported in a previous study [14].

$$Dose=\frac{factor\times MIC\times Clearance(per\;day)}{fu\times F}$$

In the abovementioned equation, clearance is expressed as L/day, the factor represents AUC_0–24h_/MIC divided by 24 h, fu represents the unbound fraction, and F represents the absolute bioavailability.

## 3. Results

### 3.1. Subsection Validation of the HPLC Method

**Figure 1:**
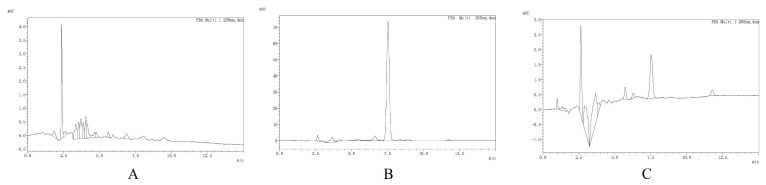
Chromatograms of cefquinome analyzed in the foal serum samples. (**A**) Chromatogram of blank serum. (**B**) Chromatogram of spiked serum. (**C**) Chromatogram of an isolated test sample containing cefquinome.

The retention time of cefquinome was determined to be 7.54 min. Its quantitative analysis demonstrated linear behavior across the concentration range of 0.02–5 μg/mL, yielding an R^2^ value of >0.99 (Figure S1). The recovery rate of cefquinome extraction from serum exceeded 80%, with a coefficient of variation of <10% being observed both throughout different runs and within the same run. The quantification and detection limits were determined to be 0.01 μg/mL and 0.005 μg/mL, respectively. Figure 1 demonstrates the results of the chromatogram.

### 3.2. Pharmacokinetic Parameters of Cefquinome in Foals

**Figure 2:**
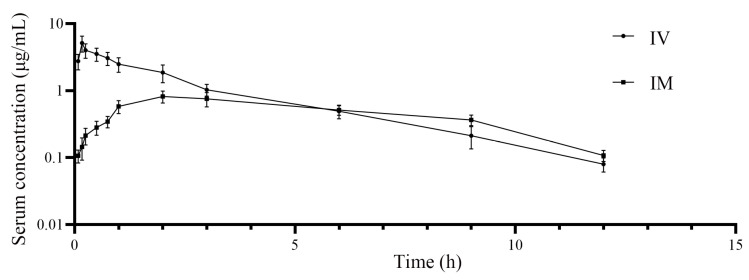
Semi-logarithmic serum concentration-versus-time graph after a single IV or IM injection of cefquinome.

Figure 2 demonstrates the curves depicting the relationship between time and cefquinome concentration in foals after IV or IM administration. Table 1 provides a summary of the PK parameters of cefquinome in the foals. After IV administration, cefquinome exhibited a shorter T_1/2β_ (2.35 h) compared to IM administration (4.16 h). The AUC_0–last_ was higher for IV administration (12.33 μg·h/mL) than for IM administration (5.41 μg·h/mL). Additionally, the MRT_0–last_ was shorter for IV administration (2.67 h) compared to IM administration (4.92 h). The CL was also lower for IV administration (0.09 L/h/kg) than for IM administration (0.15 L/h/kg). The F of cefquinome after IM administration was 43.86%.

### 3.3. In Vitro Susceptibility Testing

**Figure 3:**
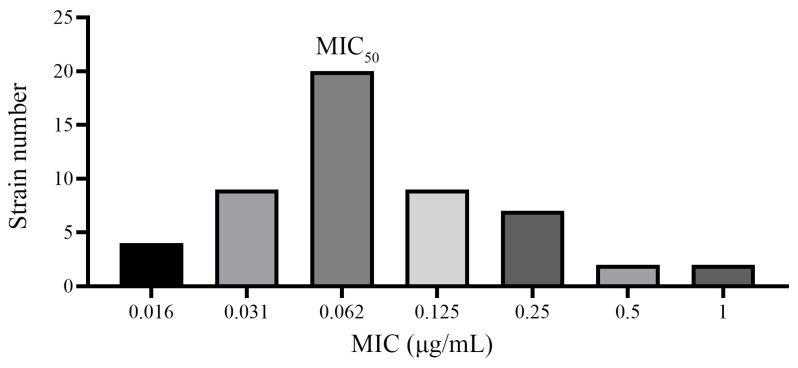
Distribution of MIC values of cefquinome for 53 *E. coli* strains.

Figure 3 demonstrates the distribution of MIC for 53 *E. coli* strains assessed for susceptibility to cefquinome in serum. MIC_50_ was found to be 0.062 μg/mL. For the quality-control *E. coli* strain ATCC25922, the reported MIC values were 0.054 μg/mL in broth and 0.031 μg/mL in serum. Table 2 shows the MIC values for 20 *E. coli* strains in both MHB and serum that had MIC values close to MIC_50._ The MIC values in MHB were approximately 1.8 times higher than those observed in foal serum. Regarding the *E. coli* strain HE13, which was isolated from a foal with septicemia, the MIC levels were determined to be 0.125 μg/mL in MHB and 0.062 μg/mL in foal serum.

### 3.4. Post-Antibiotic Effects

Table 3 demonstrates the PAEs of cefquinome at various concentrations (1×, 2×, and 4× MIC) and different exposure times (1 h and 2 h). The PAE of cefquinome remained below 0.7 h, with an increase being observed in a concentration- and duration-dependent manner.

### 3.5. In Vitro Time–Kill Curve

**Figure 4:**
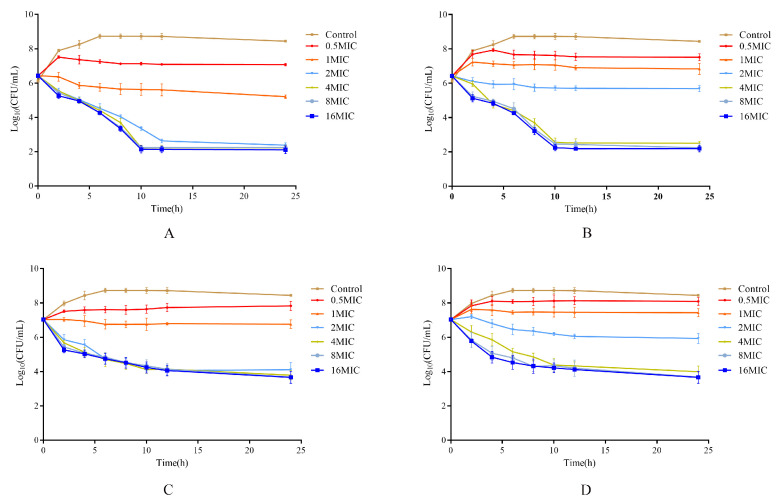
In vitro time–kill curve of cefquinome against *E. coli* ((**A**,**C**), the time–kill curve of cefquinome against *E. coli* with initial inoculum of 10^6^ and 10^7^ CFU/mL in serum; (**B**,**D**), the time–kill curve of cefquinome against *E. coli* with initial inoculum of 10^6^ and 107 CFU/mL in MHB).

Figure 4 demonstrates the in vitro time–kill curve of cefquinome against the *E. coli* strain HE13, highlighting its nature as a typical time-dependent antibiotic. The curve indicated that at an initial bacterial concentration of 10^6^ CFU/mL, cefquinome exhibited bactericidal activity at a concentration of 2× MIC (Tables S1 and S2). However, when the initial bacterial concentration was 10^7^ CFU/mL, the minimum effective concentration required to achieve bactericidal effects increased to 4× MIC (Tables S3 and S4).

### 3.6. Ex Vivo Time–Kill Curve

**Figure 5:**
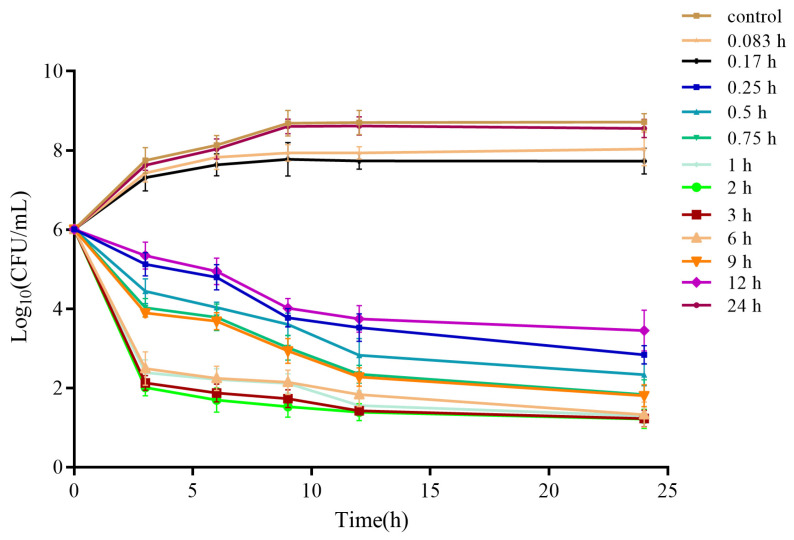
Ex vivo antibacterial activity of cefquinome against *E. coli* in serum after IM administration.

Figure 5 demonstrates the ex vivo antibacterial effect of cefquinome, highlighting its efficacy against *E. coli* over time in serum. Serum samples were obtained at designated intervals: before administration and at 0.083, 0.167, 0.25, 0.5, 0.75, 1, 2, 3, 6, 9, 12, and 24 h after IM administration. A rapid bactericidal activity was noted in serum samples collected between 0.75 h and 8 h.

### 3.7. PK/PD Modeling

**Figure 6:**
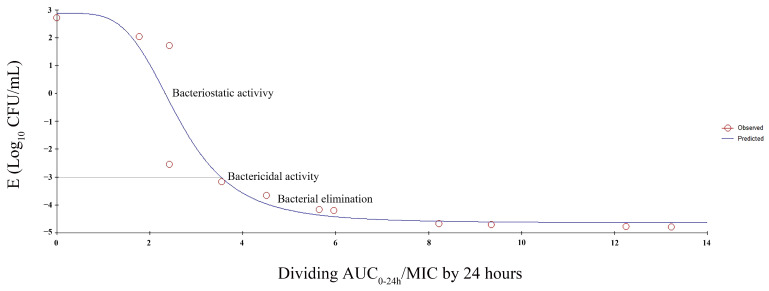
Inhibitory sigmoid E_max_ model demonstrating the relationship between the ex vivo AUC_0–24h_/MIC ratio divided by 24 h for *E. coli* in foal serum samples and bacterial counts. R^2^ denotes the correlation coefficient.

Integration of in vitro PK parameters, MIC values, and PD data from the inhibitory sigmoid Emax model provided the ex vivo AUC_0–24h_/MIC divided by 24 h values for various degrees of bacterial inhibition (Table 4). Figure 6 shows the inhibitory sigmoid Emax model depicting the relationship between ex vivo AUC_0–24h_/MIC divided by 24 h for *E. coli* in foal serum samples and bacterial counts. In the figure, the curve represents the predicted line, whereas the circles denote the mean observed values.

### 3.8. Outcomes of Monte Carlo Simulation

**Figure 7:**
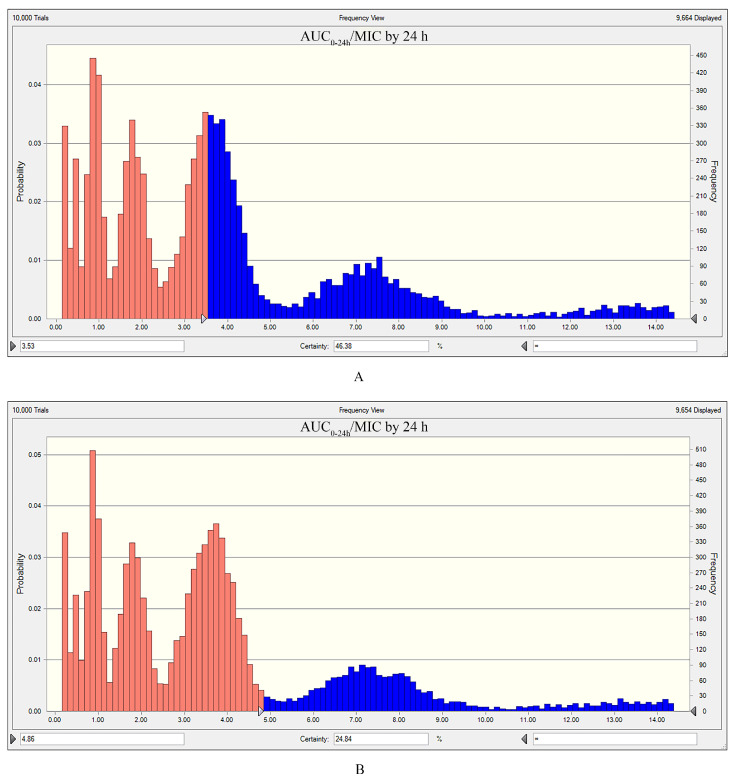
Monte Carlo simulation results. (**A**) TAR for achieving the PK/PD index targets associated with bactericidal effect. (**B**) TAR for achieving the PK/PD index targets associated with bacterial elimination. Orange indicates that the target is attained, while blue indicates that the target is not attained.

The results of the Monte Carlo simulation are shown in Figure 7. The TAR values of cefquinome at the recommended dose (1 mg/kg) against *E. coli*, under the MIC distribution of this study, required to attain the PK/PD index targets for bactericidal effect and bacterial elimination, were 46.38% and 24.48%, respectively.

### 3.9. Dose Regimen Calculation

The estimated doses required to achieve a bacteriostatic effect, a bactericidal effect, and bacterial elimination were 1.10, 1.66, and 2.28 mg/kg, respectively.

## 4. Discussion

Antibiotics are essential for both the prevention and treatment of bacterial infections. However, the inappropriate use or excessive administration of these antibiotics may result in the development and dissemination of resistant bacterial strains [15]. The spread of these resistant strains in food-producing animals poses health risks not only to livestock and poultry but also to humans [16]. Therefore, ensuring the rational use of antibiotics is crucial for minimizing the development of resistance and enhancing the effectiveness of antimicrobial agents. Integrating PK and PD parameters to predict the efficacy of antimicrobial agents and establish appropriate dosages can aid in optimizing treatment regimens [10]. The PK/PD model was used in different animal species in earlier studies [17,18]. This study focused on optimizing the dosage regimen for the treatment of septicemia caused by *E. coli*, a prevalent condition in foals.

As illustrated in the Table 5, following IV administration, the T_1/2β_ value in this study (2.35 h) was similar to that in pigs (2.33 h) but higher than those in ducklings (0.97 h), chickens (1.29 h), dogs (1.53 h), black swans (1.69 h), and premature calves (1.85 h). The AUC_0–last_ value observed in this study was 12.33 μg.h/mL, which is similar to that reported in foals by Lee [8] but exceeded that reported in horses (8.32 μg.h/mL). The CL (0.09 L/h/kg) was similar to that reported in pigs but lower than that reported in premature calves, piglets, chickens, goslings, and turkeys. Following IM administration, the C_max_ (0.89 μg/mL) was lower than in camel calves, yellow cattle, and ducks. The T_max_ (2.16 h) was longer than that observed in chickens, yellow cattle, rabbits, buffalo calves, ducks, and premature calves. The T_1/2β_ (4.16 h) was similar to that reported in premature calves, but higher than that reported in horses, sheep, yellow cattle, rabbits, and buffalo calves. The absolute bioavailability (43.86%) was lower than that in chickens, premature calves, ducks, and pigs. These differences may be attributed to variations among species. This study found that the administration route of cefquinome had a significant impact on its serum concentration and pharmacokinetic parameters. Compared to the intramuscular injection, intravenous injection had a higher AUC, which is suitable for severe infectious cases that require a rapid onset of action. However, intramuscular injection had a longer absorption half-life and mean residence time, which helps to maintain a more stable drug concentration and thus reduce the frequency of administration

Previous studies have indicated that the MIC values obtained in MHB may be higher than those obtained in similar biological fluids, potentially owing to artificial elevation [34,35]. Consequently, using MIC values derived from biological fluids is more suitable than relying on those obtained from MHB. In this study, the MIC values in MHB were 1.8 times higher than those in foal serum. The MIC values established for pathogenic organisms in MHB fail to accurately reflect the in vivo effectiveness of drugs [10]. Measuring MIC values in the corresponding biological fluid is essential, as it ensures the reliability of the integrated PK/PD model. Three MIC cut-off values are necessary to facilitate the selection of clinical breakpoints (CBPs). These values include the epidemiological cutoff (ECOFF) values and the PK/PD cutoff values defined by EUCAST and an MIC cutoff value pertinent to clinical outcomes, specifically in veterinary medicine [36]. Owing to the absence of bimodal distribution, the ECOFF value of cefquinome for *Escherichia coli* is invalid datum (https://mic.eucast.org/search/?search%5Bmethod%5D=mic&search%5Bantibiotic%5D=46&search%5Bspecies%5D=-1&search%5Bdisk_content%5D=-1&search%5Blimit%5D=50) accessed on 3 May 2024. In 308 bacterial strains tested in the VetCAST project, the MIC values for 275 strains have been reported to be 0.06 μg/mL. In this study, the MIC_50_ value was 0.062 μg/mL, which is similar to that reported in the VetCAST project. Typically, the most efficient PK/PD index for characterizing the effectiveness of time-dependent antibiotics is the proportion of time in which the drug concentration remains above the MIC (%T > MIC) [25]. For beta-lactams, %T > MIC is commonly used to predict efficacy. However, as the terminal half-life increases, the preferred index shifts towards the AUC/MIC ratio [35]. In the VetCAST project, which determined CBPs for antimicrobial susceptibility testing (AST), the AUC/MIC ratio was recommended as a universal PK/PD index for long-acting drugs. Additionally, in ex vivo PK/PD studies, it is often difficult to effectively obtain %T > MIC data [19,37]. Therefore, in this study, we adopted the AUC/MIC ratio as the PK/PD index.

The units of AUC (μg·h/mL) and MIC (μg/mL) indicate that the AUC/MIC ratio incorporates a time dimension, which can lead to confusion in clinical settings. Compared to the AUC/MIC ratio, dividing the AUC/MIC by 24 h provides a more universal metric, which can be used not only as a score but also for calculating different doses [14]. Consequently, we used the AUC_0–24h_/MIC ratio divided by 24 h as the PK/PD index in this study. The ex vivo AUC_0–24h_/MIC values divided by 24 h were evaluated, and the outcomes were integrated using the inhibitory sigmoid Emax model based on variations in bacterial counts. According to the results of the Monte Carlo simulation, the current recommended dose of 1 mg/kg achieves a target attainment rate of only 46.38% for bactericidal effects and only 24.48% for bacterial elimination effects. Based on the AUC_0–24h_/MIC ratios, the optimal doses of cefquinome required for bacteriostatic effects, bactericidal effects, and bacterial elimination in serum were 1.10, 1.66, and 2.28 mg/kg, respectively. These results show that the current recommended dose of 1 mg/kg fails to achieve the desired therapeutic effects. This phenomenon is closely related to bacterial resistance. Existing studies suggest a steady increase in cefquinome resistance across China. Xiao et al. showed that the MIC values of cefquinome against *E. coli* in the northeast region of China are gradually increasing [13]. Tong et al. indicated that feline-origin *E. coli* in different regions of China have high resistance to cefquinome (up to 85%) [38]. Mi et al. found that owing to the irrational use of drugs leading to increased MIC values for *Streptococcus suis*, the treatment rate using the recommended dose of 2 mg/kg for pigs was less than 67%. An adjustment to a dose of 3.08 mg/kg is necessary to ensure effective treatment [39]. Lee et al. used PK/PD modeling and found that the dose of cefquinome required to treat strangles caused by *Streptococcus equi* subsp. *equi* was only 0.53 mg/kg, which was closely related to the MIC value of 0.016 μg/mL [8]. The increasing global concern regarding antibiotic resistance has led to the use of PK/PD models to identify suitable medication dosages. A bactericidal dose minimizes this risk by both effectively reducing the bacterial load and balancing efficacy with prevention of resistance. In clinical practice, achieving bacterial clearance often requires prolonged or excessive doses of antibiotics, which may not be feasible due to toxicity, side effects, or patient compliance issues. Therefore, a bactericidal dose not only provides effective control of infection but also reduces the risk of antimicrobial resistance and adverse effects compared to the high dose required for complete elimination of bacteria.

This study has a few limitations that should be acknowledged. First, the infection simulated in a controlled laboratory environment did not accurately represent the natural infection. Laboratory infections are typically prompted by a single type of microorganism. In this study, the infection was caused solely by *E. coli*. However, infections in the wild are usually the culmination of interactions between multiple microbes. Second, the inherent variability among laboratory animals can yield inconsistent results, introducing an element of variability to the findings. Further investigation is warranted to address these issues. Finally, in this study, although we meticulously documented the clinical status and pharmacokinetic parameters of the foals, immune status indicators (such as white blood cell count, neutrophil count, and their percentages) were not included in the analysis. These metrics are typically used to assess the body’s immune response and infection status, and they may hold significant importance for understanding the efficacy of antibiotics and optimizing dosage regimens. Future research could consider incorporating immunological indicators, such as complete blood counts, to more comprehensively evaluate the health status and treatment outcomes of foals.

## 5. Conclusions

In this study, PK/PD modeling was used to calculate the dose of cefquinome required to treat septicemia caused by *E. coli* in foals. The results showed that the currently recommended dose of 1 mg/kg for IM injection is not effective for treatment. Owing to an increase in the MIC value, a dose of at least 1.66 mg/kg is required to effectively treat the condition. Because the therapeutic dose was determined under laboratory conditions, validating it in clinical settings is necessary to ensure therapeutic efficacy.

## Figures and Tables

**Table 1:** Pharmacokinetic parameters values of cefquinome after IV and IM administration of 1 mg/kg in foals.

Parameters (Units)	IV	IM
C_max_ (μg/mL)	—	0.89 ± 0.14
T_max_ (h)	—	2.16 ± 0.75
T_1/2β_ (h)	2.35 ± 0.38	4.16 ± 0.21
AUC_0–last_ (μg.h/mL)	12.33 ± 0.69	5.41 ± 0.81
MRT_0–last_ (h)	2.67 ± 0.13	4.92 ± 0.15
CL (L/h/kg)	0.09 ± 0.01	0.15 ± 0.02
F (%)	—	43.86 ± 5.62

**Table 2:** The MIC values of cefquinome against *E. coli* strains in MHB and foal serum.

*E. coli* Strain	MHB (μg/mL)	Serum (μg/mL)	MHB/Serum
HE01	0.125	0.062	2.0
HE02	0.125	0.062	2.0
HE03	0.1	0.062	1.6
HE04	0.108	0.062	1.7
HE05	0.125	0.062	2.0
HE06	0.1	0.062	1.6
HE07	0.125	0.062	2.0
HE08	0.108	0.062	1.7
HE09	0.108	0.062	1.7
HE10	0.1	0.062	1.6
HE11	0.108	0.062	1.7
HE12	0.125	0.062	2.0
HE13	0.125	0.062	2.0
HE14	0.1	0.062	1.6
HE15	0.1	0.062	1.6
HE16	0.108	0.062	1.7
HE17	0.1	0.062	1.6
HE18	0.108	0.062	1.7
HE19	0.125	0.062	2.0
HE20	0.125	0.062	2.0
Average	0.112	0.062	1.8

**Table 3:** Post antibiotic effects (PAEs) of cefquinome against *E. coli* HE13.

Concentration	PAE After 1 h (h)	PAE After 2 h (h)
1× MIC	0.13 ± 0.02	0.14 ± 0.03
2× MIC	0.22 ± 0.02	0.24 ± 0.03
4× MIC	0.34 ± 0.04	0.58 ± 0.05

**Table 4:** PK/PD parameter estimates for calculating AUC_0–24h_/MIC divided by 24 h to achieve various antibacterial effects.

Parameter	Value
E_max_ (Log_10_ CFU/mL)	2.88
E_0_ (Log_10_ CFU/mL)	−4.65
EC_50_	2.61
N	4.22
AUC_0–24h_/MIC divided by 24 for bacteriostatic action	2.34
AUC_0–24h_/MIC divided by 24 for bactericidal action	3.53
AUC_0–24h_/MIC divided by 24 for bacterial elimination	4.86

**Table 5:** Comparison of pharmacokinetic parameters after IV and IM administration across different animal species.

**Route**	**IV**										
**Parameter**	**Species**										
	**Foals**	**Pigs**	**Ducklings**	**Goslings**	**Chickens**	**Dogs**	**Black Swans**	**Premature Calves**	**Horses**	**Piglets**	**Turkeys**
T_1/2β_ (h)	2.77	2.33	0.97	1.73	1.29	1.53	1.69	1.85	2.77	1.85	1.56
AUC_0–last_ (μg·h/mL)	15.15	21.28	6.25	4.39	5.33	5.15	16.5	15.74	8.32	8.07	6.22
CL (L/h/kg)	0.06	0.09	0.32	0.45	0.35	0.49	0.13	0.13	0.12	0.26	0.32
Reference	[8]	[19]	[20]	[20]	[21]	[22]	[23]	[24]	[25]	[26]	[27]
**Route**	**IM**										
**Parameter**	**Species**										
	**Camel Calves**	**Pigs**	**Yellow Cattle**	**Ducks**	**Chickens**	**Rabbits**	**Buffalo Calves**	**Premature Calves**	**Sheep**		
C_max_ (μg/mL)	28.40	6.15	2.34	2.37	3.04	6.93	6.93	4.56	2.60		
T_max_ (h)	0.42	0.34	0.78	0.30	0.25	0.33	0.50	1.00	0.50		
T_1/2β_ (h)	17.40	2.30	2.78	0.66	1.35	0.72	3.73	4.74	1.88		
F (%)	—	103.88	104.00	—	95.81	—	86.30	141.22	89.31		
Reference	[28]	[19]	[29]	[30]	[21]	[31]	[32]	[24]	[33]		

## Data Availability

The authors confirm that the data supporting the findings of this study are available within the article.

## Supplementary Materials

The following supporting information can be downloaded at: https://www.mdpi.com/article/10.3390/vetsci12040294/s1, Figure S1: Calibration curve for cefquinome quantification via HPLC; Table S1: The data regarding in vitro time–kill curve in MHB at the initial concentration of 10^6^ CFU/mL; Table S2: The data regarding in vitro time–kill curve in serum at the initial concentration of 10^6^ CFU/mL; Table S3: The data regarding in vitro time–kill curve in MHB at the initial concentration of 10^7^ CFU/mL; Table S4: The data regarding in vitro time–kill curve in serum at the initial concentration of 10^7^ CFU/mL.
