# Supplementary material for: Ex Vivo Pharmacokinetic/Pharmacodynamic Integration Model of Cefquinome Against Escherichia coli in Foals
Source: Vet Sci. 2025 Mar 22;12(4):294. doi: 10.3390/vetsci12040294 (PMC12031376; doi:10.3390/vetsci12040294)
Supplement: Supplementary file 1 [file vetsci-12-00294-s001.zip › Table S1.pdf]

Table S1: *In vitro* time-kill curve in MHB at the initial concentration of 10^6 CFU/mL

Time (h)	the density of the *Escherichia coli* (log_{10}CFU/mL)						
	**Control**	**0.5×MIC**	**1×MIC**	**2×MIC**	**4×MIC**	**8×MIC**	**16×MIC**
0	6.00	6.00	6.00	6.00	6.00	6.00	6.00·
2	7.89	7.69	7.23	6.11	5.96	5.24	5.13
4	8.25	7.93	7.13	5.93	4.77	4.94	4.83
6	8.53	7.67	7.06	5.94	4.40	4.51	4.26
8	8.57	7.65	7.09	5.74	3.69	3.33	3.20
10	8.62	7.62	7.06	5.72	2.56	2.45	2.25
12	8.66	7.54	6.90	5.70	2.53	2.43	2.18
24	8.44	7.52	6.83	5.68	2.51	2.24	2.19
