# Supplementary material for: Ex Vivo Pharmacokinetic/Pharmacodynamic Integration Model of Cefquinome Against Escherichia coli in Foals
Source: Vet Sci. 2025 Mar 22;12(4):294. doi: 10.3390/vetsci12040294 (PMC12031376; doi:10.3390/vetsci12040294)
Supplement: Supplementary file 1 [file vetsci-12-00294-s001.zip › Table S2.pdf]

Table S2: *In vitro* time-kill curve in MHB at the initial concentration of 10^7 CFU/mL.

Time (h)	the density of the *Escherichia coli* (log_{10}CFU/mL)						
	**Control**	**0.5×MIC**	**1×MIC**	**2×MIC**	**4×MIC**	**8×MIC**	**16×MIC**
0	7.00	7.00	7.00	7.00	7.00	7.00	7.00
2	7.97	7.83	7.63	7.20	6.30	5.83	5.78
4	8.43	8.10	7.59	6.79	5.85	5.07	4.83
6	8.53	8.07	7.45	6.45	5.15	4.80	4.53
8	8.58	8.09	7.48	6.35	4.85	4.31	4.32
10	8.63	8.12	7.39	6.19	4.37	4.33	4.21
12	8.65	8.13	7.37	6.05	4.33	4.20	4.11
24	8.44	8.09	7.33	5.93	3.98	3.68	3.65
