# Supplementary material for: Ex Vivo Pharmacokinetic/Pharmacodynamic Integration Model of Cefquinome Against Escherichia coli in Foals
Source: Vet Sci. 2025 Mar 22;12(4):294. doi: 10.3390/vetsci12040294 (PMC12031376; doi:10.3390/vetsci12040294)
Supplement: Supplementary file 1 [file vetsci-12-00294-s001.zip › Table S3.pdf]

Table S3: *In vitro* time-kill curve in serum at the initial concentration of 10^6 CFU/mL

Time (h)	the density of the *Escherichia coli* (log_{10}CFU/mL)						
	**Control**	**0.5×MIC**	**1×MIC**	**2×MIC**	**4×MIC**	**8×MIC**	**16×MIC**
0	6.00	6.00	6.00	6.00	6.00	6.00	6.00
2	7.89	7.51	6.36	5.53	5.55	5.43	5.26
4	8.25	7.36	5.86	5.03	4.96	4.94	4.95
6	8.53	7.25	5.75	4.53	4.40	4.26	4.26
8	8.57	7.12	5.65	4.03	3.69	3.41	3.34
10	8.62	7.13	5.63	3.35	2.23	2.24	2.21
12	8.66	7.09	5.61	2.62	2.21	2.23	2.19
24	8.44	7.07	5.21	2.38	2.20	2.21	2.17
