# Supplementary material for: Ex Vivo Pharmacokinetic/Pharmacodynamic Integration Model of Cefquinome Against Escherichia coli in Foals
Source: Vet Sci. 2025 Mar 22;12(4):294. doi: 10.3390/vetsci12040294 (PMC12031376; doi:10.3390/vetsci12040294)
Supplement: Supplementary file 1 [file vetsci-12-00294-s001.zip › Table S4.pdf]

Table S4: *In vitro* time-kill curve in serum at the initial concentration of 10^7 CFU/mL

Time (h)	the density of the *Escherichia coli* (log_{10}CFU/mL)						
	**Control**	**0.5×MIC**	**1×MIC**	**2×MIC**	**4×MIC**	**8×MIC**	**16×MIC**
0	7.00	7.00	7.03	7.00	7.00	7.00	7.00
2	7.97	7.51	7.04	5.85	5.67	5.43	5.26
4	8.43	7.59	6.95	5.56	5.13	5.07	5.03
6	8.53	7.61	6.75	4.75	4.70	4.83	4.75
8	8.58	7.59	6.68	4.55	4.47	4.53	4.51
10	8.63	7.63	6.65	4.25	4.12	4.33	4.24
12	8.65	7.73	6.67	4.05	4.14	4.13	4.05
24	8.44	7.83	6.63	4.11	3.79	3.68	3.65
