# Supplementary figures and images for: Ex Vivo Pharmacokinetic/Pharmacodynamic Integration Model of Cefquinome Against Escherichia coli in Foals
Source: Vet Sci. 2025 Mar 22;12(4):294. doi: 10.3390/vetsci12040294 (PMC12031376; doi:10.3390/vetsci12040294)

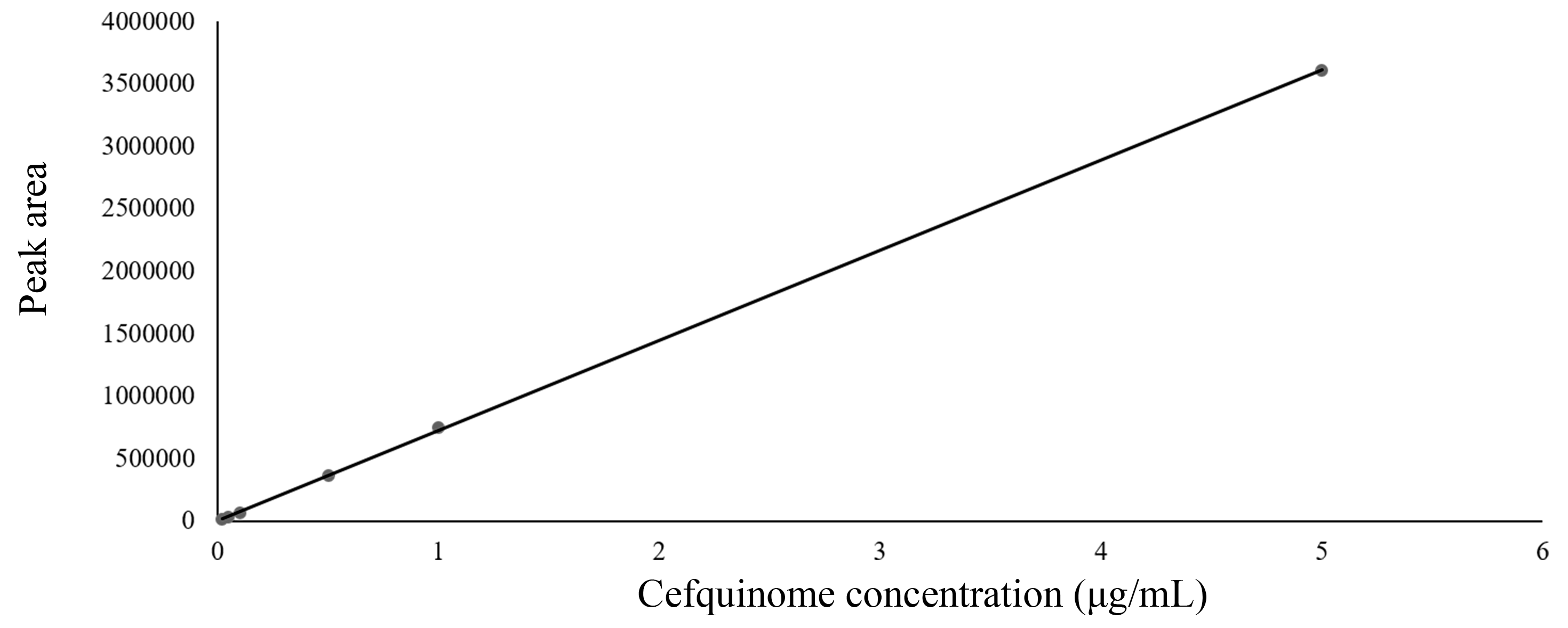

Supplement: Supplementary file 1 [file vetsci-12-00294-s001.zip › Figure S1.tif]
